# Temperature directly correlates with emergency surgical case admissions independent of seasonality

**DOI:** 10.1038/s41598-025-00957-9

**Published:** 2025-05-06

**Authors:** Adrian Patenge, Dominik Pförringer, Nicole Estrella, Annette Menzel, Konstantin Krüger, Leo Richter, Lisa Schmid, Michael Dommasch, Aaron Becker von Rose

**Affiliations:** 1 Klinikum Rechts Der Isar, Emergency Department, Technical University of Munich, Munich, Germany; 2 Klinikum Rechts Der Isar, Department of Trauma Surgery, Technical University of Munich, Munich, Germany; 3 Ecoclimatology, TUM School of Life Sciences, Technical University of Munich, Freising, Germany; 4 Institute for Advanced Study, Technical University of Munich, Garching, Germany; 5 Emergency Department, University Hospital Rechts Der Isar, Technical University Munich, Munich, Germany

**Keywords:** Temperature, Overcrowding, Weather, Emergency medical services, Emergency department, Environmental sciences, Environmental social sciences, Natural hazards, Diseases, Health care, Health occupations, Medical research, Risk factors

## Abstract

Weather parameters impact patient admission to emergency departments (EDs) and due to pressures of overcrowding, it is imperative to better forecast admission levels. This retrospective single-centre study aimed to assess whether ambient temperature independently predicts emergency department (ED) admissions, beyond typical seasonal variation. We analysed 150,751 admissions to the ED of the University Hospital Klinikum rechts der Isar (MRI) in Munich, Germany from 2019–2022. Patients were divided into either surgical or internal medicine groups. Their post-treatment status as well as discharge type (‘outpatient’ versus ‘inpatient’) was also recorded. Descriptive statistics as well as linear models were used to identify and test statistically significant correlations of ED and weather variables. Patient admissions of the surgery group were directly correlated with changes in ambient temperature. This relationship persisted consistently across all seasons, suggesting a temperature effect that is independent of typical seasonal fluctuations. Whilst ED patient intake of the internal medicine group decreased during some holidays, spring as well as on weekends, sharp increases could be seen during the Oktoberfest period and Christmas. Despite minor variances, this was not the case in the surgery group. Here, the overall direct correlation of temperature and surgical patient levels for the period between 2019–22 seems highly significant. Temperature changes lead to more surgical cases irrespective of the season and more outpatient discharges, whilst inpatient admissions generally seem rather unresponsive to weather changes. A better allocation of resources in ED departments results from superior understandings of trigger factors and temperature has thus been rendered a particularly potent one.

## Introduction

Daily patient arrivals at emergency departments (EDs) remain highly unpredictable with overall increases in demand detectable globally^1^. Surges in demand present a significant contributing factor for hospital overcrowding, argued to be one of the most principal limiting factors to timely and efficient treatment in health care today^2^. Longer waiting times, perceptions of poorer healthcare overall besides the actual worsening of patient outcomes result and patients might even leave prior to any assessment. Notable increases in ED admission levels have been linked to factors such as weekends, bank holidays and seasons^3,4^ or indeed major events involving excessive alcohol consumption^5^, yet environmental factors impact too. Climate change, unequivocal as it is, has been shown to affect human health significantly and whilst manifold and not always quantifiable in the extent of health effects, extreme weather events such as heat waves or flooding have extensively been linked to increased heat-related illnesses and deaths^6^, higher frequencies of vector-borne diseases such as Malaria^7^ or the exacerbation of chronic conditions such as COPD^8^,^9^. Less research – however – focuses on the effects of ambient temperature generally, outside of extreme events or specific medical conditions.

Key questions asked revolved around whether only ‘extreme’ temperatures led to spikes in ED patient admissions or whether indeed comprehensive trends could be observed. Further, all patient subgroups (surgery versus internal medicine) were tested for differences in their respective association with weather variables. Only few studies have investigated how environmental variables affect overall patient admissions, irrespective of general mortality or specific health conditions^10^. The ED often presents the first point of contact for incoming hospital patients and a better understanding of contributing factors leading to changes in patient admission levels might allow for better forecasting models to be implemented. While prior studies have highlighted links between weather extremes and health outcomes, most focus on specific diagnoses, vulnerable populations, or extreme events. Few have investigated general ED admission trends across a broad urban population or attempted to control for seasonal effects when evaluating the role of ambient temperature. This study addresses this gap by analysing over 150,000 ED admissions in Munich over four years, with a focus on identifying whether temperature predicts admission patterns independently of seasonal variation^11^. Despite challenges of multi-dimensionality and possible confounding between different environmental variables remaining, the observable correlation between ambient temperature overall as well as ED admission levels, especially in the surgery patient group, stands. While causality cannot be inferred, the consistency of the association across multiple years is noteworthy. Should the effects of climate change lead to significant temperature increases overall, better forecasting models of patient admissions might alleviate what is increasingly becoming the major problem facing health systems globally: the “imbalance of the need for emergency care and the hospital’s availability to provide [this] service”^12^.

## Patients and methods

### Emergency department data

To investigate and analyse emergency department admissions, we examined a comprehensive dataset of patient admission records from the central emergency department at the University Hospital Klinikum rechts der Isar (MRI) in the Haidhausen district of central Munich, Germany. The MRI is one of two University Hospitals in Munich and holds its largest emergency department with over 37,000 patient admissions on average annually. Our dataset spans four years, from 2019 to 2022, and includes electronic medical records from 150,751 patient admissions. The 4-year daily data include age, sex, admission type (self-admission versus via ambulance-service), categorized department (trauma / surgery versus internal medicine versus other departments) after triage as well as the status post treatment in the ED (admission to ward versus same-day discharge). We grouped cases of abdominal as well as general surgery with trauma surgery cases. Furthermore, all patients admitted for further treatment on a ward or those being transferred to other medical facilities were grouped. All data were synopsised for every calendar in the period considered to allow for proper comparison with weather variables. A full list of all recorded categories besides baseline and clinical characteristics can be found in Table 1.

**Table 1:** Baseline and clinical characteristics for all 150,751 patient admissions at the emergency department at the University Hospital Klinikum rechts der Isar (MRI) for the period from 2019 to 2022.

Category		Surgery	Internal medicine	Other departments
Total number of patients		73,401	52,082	25,268
Gender	Male	39,446	26,621	11,968
	Female	33,955	25,460	13,301
Age	0—18	3536	703	531
	19—50	38,055	22,288	13,776
	51—70	16,395	13,960	6236
	71—80	7516	7974	2824
	> 80	7899	7157	1901
Urgency level	red	1095	525	101
	orange & yellow	20,165	29,883	11,197
	blue & green	52,141	21,674	13,970
Discharge	inpatient	10,710	18,448	8256
	outpatient	62,601	33,478	16,989
	death	57	133	8
	other	33	23	15
Admission	self-admission	51,730	36,672	17,690
	ambulance service	21,671	15,410	7578

The study was performed in agreement with the Declaration of Helsinki and approved by the local ethics committee (Ethikkommission University Hospital rechts der Isar, Technical University Munich, Germany: 2023–682-S-CB). Patient data were completely pseudonymized for further analysis and thus no separate Consent to Participate declarations were required from individual patients. Data are reported in accordance with STROBE criteria.

### Environmental data

Hourly weather data for 2019–2022 for precipitation levels in mm (litre per m^2^) as well as temperature in degrees Celsius (°C) were downloaded from Climate Data Center of the German National Meteorological Service, the Deutscher Wetterdienst (DWD, 2023) and refer to measurements taken at the weather station ‘Munich City—Neuhausen’ (Station ID 3379) at a height of 515 m above sea level. We obtained data on ambient air pollution from the Bavarian State Office for Environment (2023) as hourly concentration measurements (μg/m^3^) of particulate matter (PM) with aerodynamic diameter of less than 10 μm (PM10), nitrogen dioxide (NO_2_) and ozone (O_3_). Air pressure data (in hPa) and data on general visibility (in metres) were also provided. Missing values were identified in less than 0.3% of cases, with none being observable in the case of temperature or precipitation. The median of the immediate neighbouring values, specifically three hours prior and after was used to fill gaps. In cases of longer periods of error (any two hours consecutive or more), the respective 24-h median before and after was used to approximate missing data. Hourly data was subsequently aggregated and the median, in relevant cases also maximum and minimum per calendar day calculated.

### Statistical methods

Environmental and emergency department (ED) data were aggregated to daily intervals, providing a uniform basis for analysis. Patient characteristics were summarized and presented in a descriptive summary table based on predefined categories of the ED administrative system. Utilizing Python for all computational and statistical procedures, an initial Pearson correlation matrix identified potential dependencies among variables. This preliminary analysis directed our focus toward ambient temperature as one key impact variable of patient admissions. Illustrating this, admission numbers of both surgical and internal medicine patients were then plotted against the median ambient temperature over the span of one calendar year, with the time adjusted to calendar weeks and the respective four-year median values selected for each variable. This approach allowed for seasonal fluctuations to be levelled out by aggregating data across the same calendar weeks for four consecutive years. The confounding effect of seasonal patterns could thus be reduced to allow for a more focused analysis of temperature as a more general independent variable. A polynomial regression analysis ensued to further explore the anticipated relationship between median daily ambient temperature and the median number of surgical case admissions, with the results displayed alongside 95% confidence intervals and odds ratios for a more comprehensive understanding. This approach allowed for the modelling of more complex relationships than a simple linear regression, particularly when the effect of temperature on admissions is not expected to be strictly linear. The degree of interdependence and correlation between variables was comprehensively illustrated using a forest plot. This visual tool facilitated the simultaneous comparison of multiple weather variables, with odds ratios and 95% confidence intervals clearly displayed. The forest plot effectively summarized these findings.

## Results

In the duration between 2019–2022, a total number 150,751 patients were admitted to the ED at the University Hospital Klinikum rechts der Isar (MRI). Of all patients, 78,035 (51.76%) were male and 72,716 (48.24%) female. Outpatient discharges – patients leaving for home following treatment – totalled 113,068 (75%), inpatient discharges and patients being admitted to wards post-ED treatment amounted to 37,414 cases (24.82%) besides 198 (0.13%) recorded deaths and 71 (0.05%) ‘other’ discharges (including patients leaving prior to any assessment amongst others). Overall, median age was 49 years (Interquartile Range [IQR]: 30; 68]). With a primary focus on adult care, only 4,770 (3.16%) of patients admitted were in the age group of 0–18 years, 74,119 (49.17%) in the 19–50 years group, 36,591 (24.27%) in the 51–70 years group, 18,314 (12.15%) in the 71–80 age group and 16,957 (11.25%) in the 80 + years group. Whilst overall patient admission for younger ages was significantly higher in the surgery group when compared to the internal medicine group, this equalled out gradually with increasing patient age. This effect can be seen in the bar charts in Supplementary Figs. 1–6. Only 1,721 (1.14%) patients admitted fulfilled Manchester Triage category ‘Red’, with 61,245 (40.63%) classified as ‘Orange / Yellow’ and the majority with 87,785 (58.23%) grouped in the ‘Blue / Green’ Triage category. A majority of 106,092 (70.38%) patients presented themselves independently for admission at the ED, whilst 44,659 (29.62%) cases were transferred for admission using the ambulance services. The summarised data above can be viewed in Table 1.

**Fig. 1:**
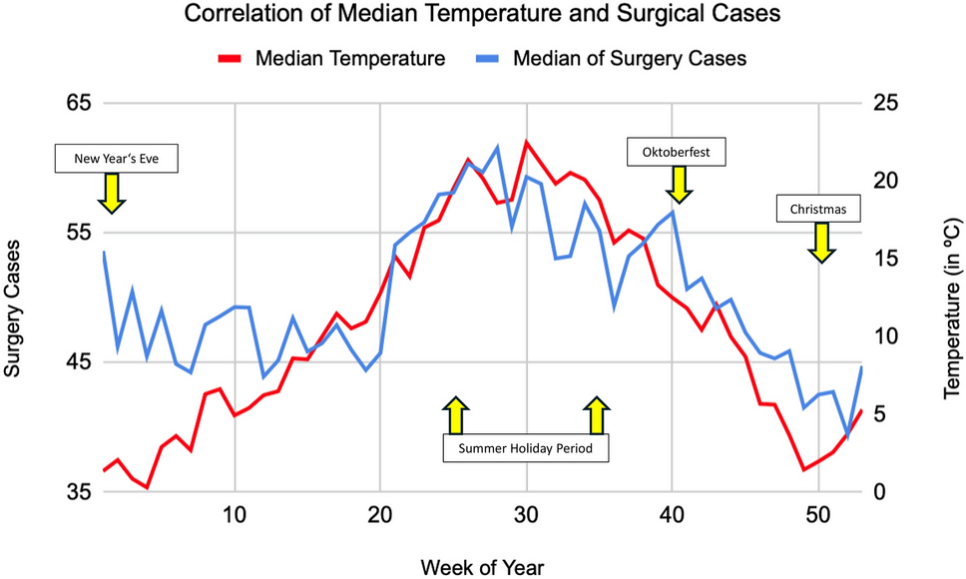
Graph showing the median of surgical case admissions in absolute numbers plotted against the median of ambient temperature in Celsius (°C). Medians were calculated by calendar week over four years (2019–2022) to control for seasonal variation, isolating temperature effects.

**Fig. 2:**
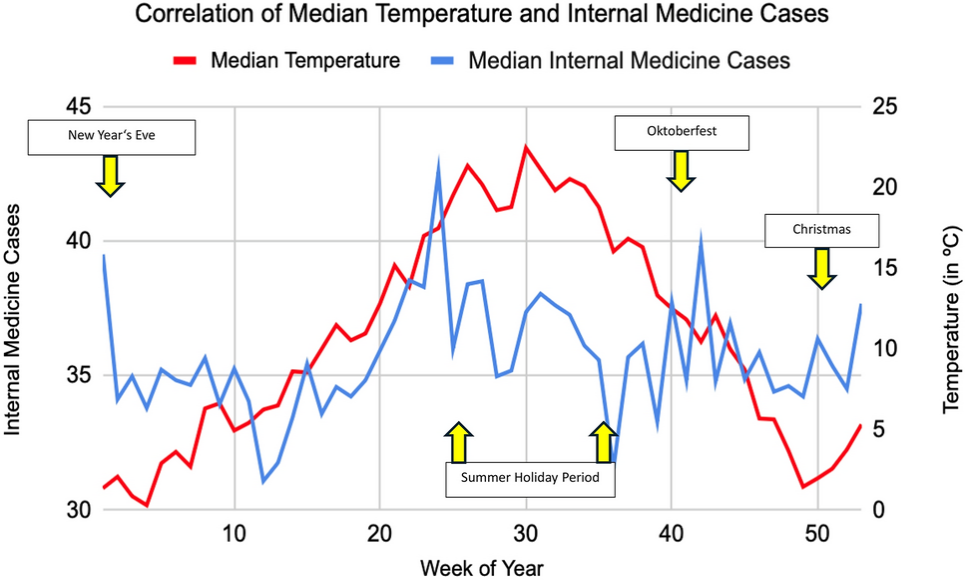
Graph showing the median of internal medicine case admissions in absolute numbers plotted against the median of ambient temperature in Celsius (°C). Medians were calculated by calendar week over four years (2019–2022) to control for seasonal variation, isolating temperature effects.

**Fig. 3:**
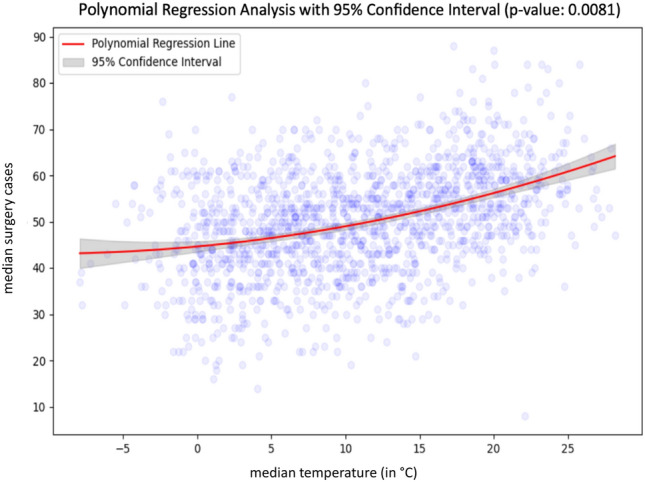
Polynomial regression plot of median daily ambient temperature and median surgical case admissions.

**Fig. 4:**
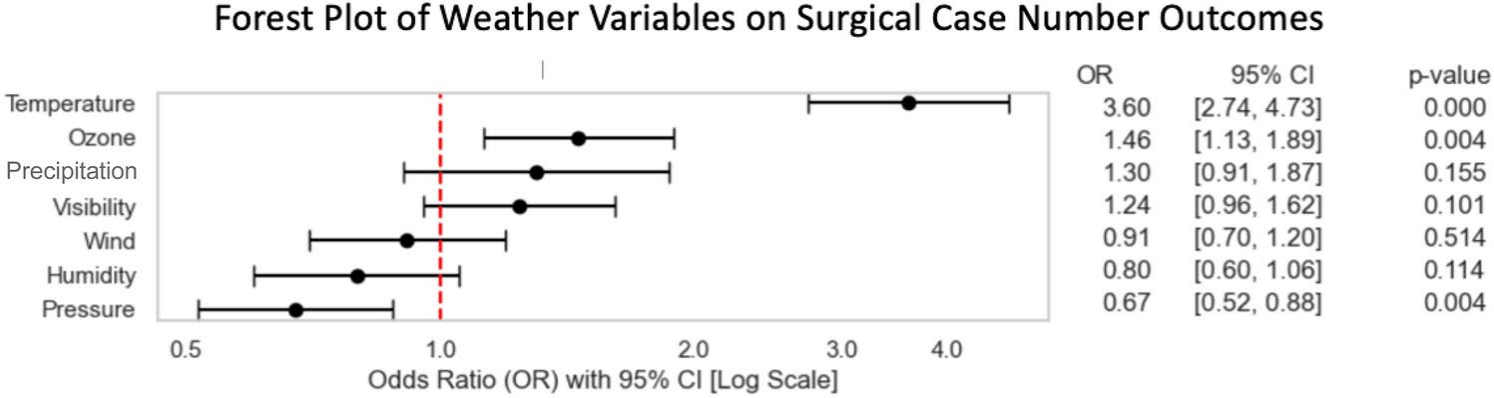
Forest plot showcasing the likelihood of a given weather variable impacting on the number of surgical patient group admissions.

Following initial correlation analyses, ambient air temperature showed most correlative potential, with *r* values of 0.39 in a classic Pearson correlation matrix when comparing ambient temperature and Surgical Group cases as well as 0.16 when considering the internal medicine group exemplarily. Other environmental variables showed only limited indications for linear relationships. A summary correlation matrix showing all selected variables can be found in the Supplementary Fig. 7. Focus thus lay on ambient temperature as the principal environmental variable to be considered and Fig. 1 and Fig. 2 show aggregated data for both ambient data and the number of patients admitted to the ED over the period from 2019 to 2022 and individual calendar weeks for both the surgical and internal medicine patient group. A first indication of a correlation can be identified in the case of the surgical group, with rising temperatures almost matching increases in ED admissions, except for the winter season in the first calendar weeks. This observation is less striking when considering admissions of the internal medicine group. Whilst equally associated to a degree, a stark contrast of a drop in cases despite rising ambient temperatures is observable in the summer period. Spikes around the time of the Oktoberfest and New Year’s Eve exemplarily are identifiable in both the surgical and internal medicine group. These fluctuations are likely influenced by temporary changes in the city’s population. Munich is a major tourist and conference destination, and the population served by the interdisciplinary ED at the MRI can vary considerably depending on the time of year. Notably, during Oktoberfest, Munich’s population temporarily increases by approximately 1.5 million visitors over a two-week period^5^. Similar surges, although smaller, are expected during the Christmas season and public holidays. Unfortunately, reliable weekly population estimates or tourist numbers at a daily resolution were not available for direct comparison, but these trends likely contribute to the observed spikes in ED utilisation. Importantly, however, the overall correlation between temperature and surgical admissions remained consistent even when excluding these known high-volume periods. Following the observed association of ambient temperatures and admission numbers, especially in the surgical group, polynomial regression analyses solidified an assumed correlation between the variables. With a p-value of 0.0081 and an odds ratio (OR) of 3.60, median ambient temperatures appeared to directly affect daily ED admission numbers for patients of the surgical group and with rising temperatures generally, an increase in patient admission to the ED is to be expected as well. This highly significant correlation is shown in Fig. 3 with included 95% confidence intervals as well as the number of total patient admissions in the surgical group (73,041) analysed shown as graphical dots. Comparing the ORs of other environmental variables to that of ambient temperature in the case of surgical group patient admission, the forest plot of Fig. 4 shows no other environmental variable to have a similarly robust effect.

## Discussion

Environmental variables amongst others directly affect patient admission levels at EDs worldwide, as has been widely postulated^5,13^ . Given the increasing strain on ED capacity, identifying correlates of patient admission levels may support the development of improved forecasting models.^2^. This retrospective study analysed over 150,751 patient admissions at a high-volume ED in the German city of Munich between 2019 to 2022 as well as corresponding environmental variables for that same period. It presents a comprehensive investigation of a large cohort of patients besides a coherent set of weather data with several specific variables. Whilst clearly complex, it seems important to already accept that no single environmental variable alone can stand to explain variance in admission levels at EDs^14^. Indeed, weather per se seems to imply the interplay of a multitude of independent factors. Variables affecting ED patient traffic must therefore be equally manifold, highly interdependent, and in the end only partially influenced by weather^5^. Yet, considering that outside of extreme weather events and specific medical conditions almost no publication sought to determine general trends as well as comprehensive patterns, this investigation achieved just that. It clearly shows that already minor increases of ambient temperatures outside the range of extremes can have a linear and direct relationship with ED patient admissions. This agrees with numerous other studies in principle, even though most focus on either extreme weather phenomena, specific age groups or distinct medical conditions^15–18^. Our study expands on prior work by demonstrating that even moderate, non-extreme changes in ambient temperature are associated with changes in overall surgical ED admissions in a general adult urban population. Importantly, this association held consistently across the year after controlling for seasonality. Few studies in temperate European or international settings have systematically analysed such effects at this scale while accounting for seasonal variation. Exemplarily, Bernstein et al.^15^ highlighted how increased temperatures led to overall increases in children ED admissions across the United States while Qu^16^ focused on kidney disease and how temperature increases led to exacerbations of symptoms and thus higher rates of hospitalization in New York. Higher temperatures were also shown to precipitate increases in Asthma-related ED admissions in Taiwan^17^ and similarly presented a potent trigger for a significant increase of cardio-respiratory ED admissions in Bangladesh^18^. Of course, other weather variables have been equally shown to have an effect. Whether it be air pollution’ trigger effect on higher ED admissions for CNS associated diseases^19^ or the impact of ozone level increases on respiratory conditions in the greater Washington area and ensuing higher ED admission rates^20^: weather undoubtedly shapes ED patient admissions and temperature as a variable may therefore be seen as *one* valid predictor variable for ED admission levels.

In the specific context of this investigation, patients’ admissions of the surgical group increased almost parallel in scope to the increase in ambient temperature and similar, yet weaker trends were observable in the internal medicine group. External and extraordinary factors such as school holidays or indeed the Oktoberfest, led to generally smaller alterations to surgical group patient admissions than in the internal medicine group, however not defying the overall observation of a linear and positive relationship. Importantly, the persistence of this relationship across all seasons—as shown using multi-year calendar week medians—confirms that the effect of temperature on surgical admissions is not merely a reflection of seasonal patterns. By aggregating the same periods across multiple years, our design effectively filtered out predictable calendar effects (such as summer holidays or the Oktoberfest season).

The summer holiday period exemplarily led to strong negative corrections in the ED admission figures in the internal medicine patient group, which however could not be observed in the surgical patient group. This is illustrated in Figs. 1 and 2. Positive corrections above associated median admission numbers were observable in the period surrounding the 40-calendar week and the Oktoberfest generally^5^. Trigger effects with increases in ED admissions were identifiable in both groups and correspond with an overwhelming increase in Munich population levels for the mentioned period. With increasing age, fewer notable fluctuations were detectable as elderly patients generally seem to be more independent of above factors, again see Supplementary Figs. 1–6. Age as a variable generally also mattered less in the context of the internal medicine group. Irrespective of age group, limited patterns were observable, even if admissions of a specific age group were correlated with ambient temperature as weather variable. This was quite different when considering the surgery group. Here, especially the age group 19–50 showed the most significant result and strongest correlation. Besides weekends that are regarded as having generally fewer ED admissions^21^, seasonal effects with overall increases in patient numbers in summer when compared to the winter period or abnormalities in median ED admission levels in the context of specific bank holidays^5^, ambient temperature has been shown to be independently associated with ED patient admission levels. Other environmental factors in comparison seemed to have no or almost no effect when considered singularly. Even moderate ambient temperature changes showed significant impact, thus extending beyond just extreme weather events. With overall ambient temperature increases predicted to become even more dynamic in the wake of potentially accelerating global climate change^22^, temperature may serve as a relevant variable in predictive modelling of ED demand. However, further research is needed to evaluate its practical application in operational settings.

## Limitations

Observed associations and statistically derived linear regression do not automatically imply causality. The retrospective identification of the study cohort constitutes an inherent bias. Whilst increases in ambient temperature certainly correlate with increases in ED patient admissions, other weather factors, not within the narrow scope of this investigation, most certainly have an effect too^19,20^. Besides mere higher temperatures, climate change has equally been linked to worsening air pollution levels, changes to precipitation patterns or humidity amongst others. The specific effect of these variables singularly besides their interplay with ambient temperature has not been considered in depth, outside of an initial correlation matrix. Especially this interplay of weather variables with potentially reinforcing effects on patient admission levels should be studied further. Urban areas might also show different weather patterns than more rural areas or indeed locations with significantly different climates to the location of this investigation. Assumptions on weather developments as well as their implications of hospitalizations may therefore be limited too. Finally, while the observed associations are statistically robust, the retrospective and observational nature of the study limits causal inference. The data do not permit conclusions about the effectiveness of operational changes, such as staffing adjustments, and any such implications remain speculative. Prospective studies are needed to assess the practical value of incorporating temperature trends into ED planning. General methodological limitations associated with retrospective study designs also apply.

## Conclusion

Increases in ambient temperature have been shown to directly correspond with increases in ED patient admission levels at the ED of the University Hospital Klinikum rechts der Isar (MRI). This is especially the case when considering admissions of the surgical patient group, where a correlation seems most apparent. Even modest changes in ambient temperatures seem to have a notable effect on ED patient admission levels, even though this is less applicable to elderly patients overall. This association remained robust after controlling for seasonal factors, further strengthening the case for temperature as an independent predictor of emergency demand. As climate change is expected to lead to further increases in median temperatures globally, recognising the positive and linear association between temperature and ED patient admissions may help inform future forecasting models. While this could support operational planning, further prospective research would be required to evaluate its impact on overcrowding directly.

## Supplementary Information

Supplementary Information.

## Data Availability

The data that support the findings of this study are available from the German Meteorological Service (DWD) as well as the Bavarian State Office for Environment (weather data) and the Central Emergency Department of the University Hospital Rechts der Isar (MRI) at the Techncial University of Munich (clinical data). Restrictions apply to the availability of these data, which were used under license for the current study, and so are not publicly available. Data are however available from the corresponding authors upon reasonable request and with permission of the aforementioned parties.
