# Supplementary material for: Temperature directly correlates with emergency surgical case admissions independent of seasonality
Source: Sci Rep. 2025 May 6;15:15832. doi: 10.1038/s41598-025-00957-9 (PMC12055994; doi:10.1038/s41598-025-00957-9)
Supplement: Supplementary file 1 — Supplementary Information. [file 41598_2025_957_MOESM1_ESM.pptx]

## Slide 1
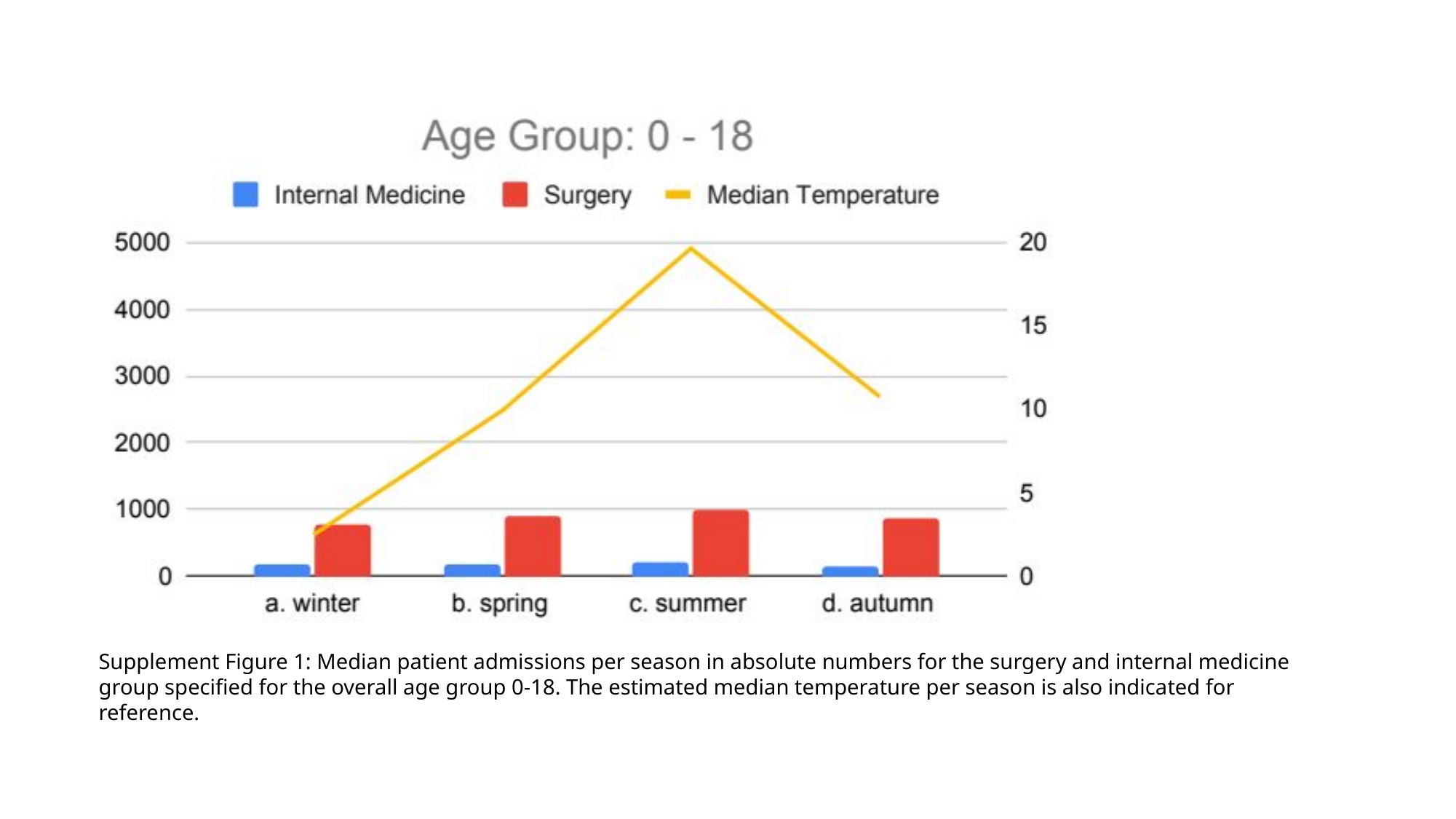

Supplement Figure 1: Median patient admissions per season in absolute numbers for the surgery and internal medicine group specified for the overall age group 0-18. The estimated median temperature per season is also indicated for reference.

## Slide 2
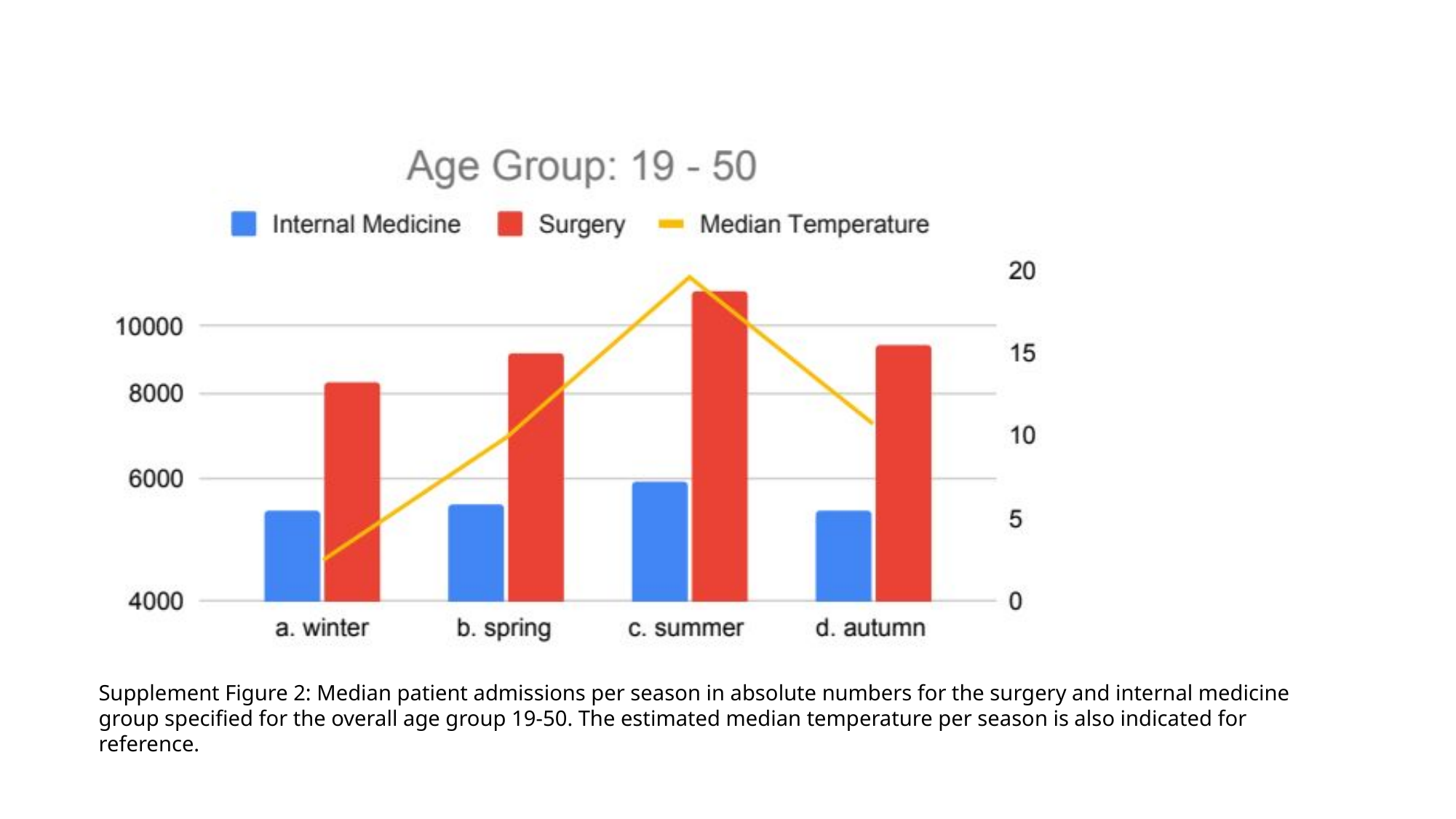

Supplement Figure 2: Median patient admissions per season in absolute numbers for the surgery and internal medicine group specified for the overall age group 19-50. The estimated median temperature per season is also indicated for reference.

## Slide 3
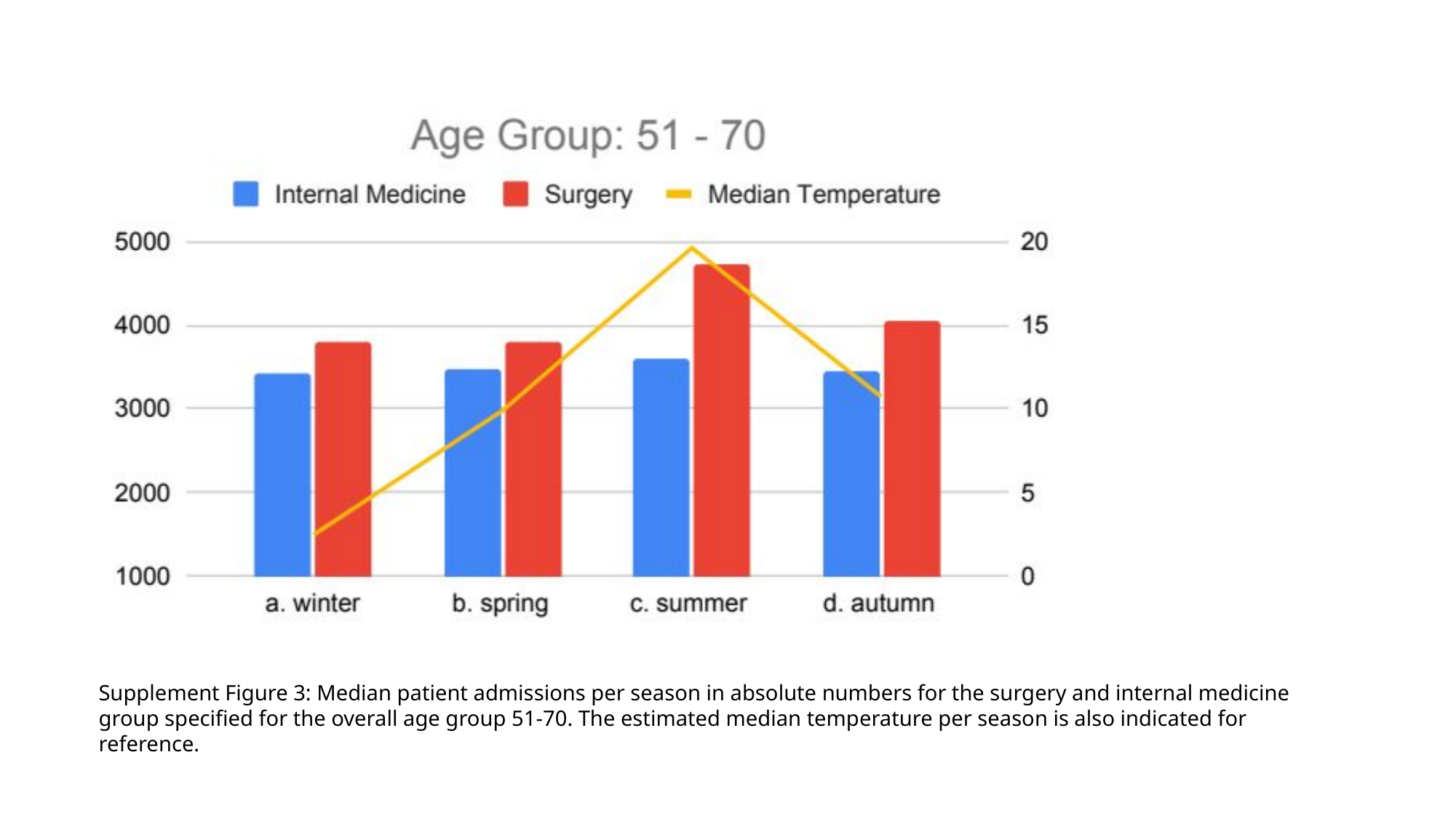

Supplement Figure 3: Median patient admissions per season in absolute numbers for the surgery and internal medicine group specified for the overall age group 51-70. The estimated median temperature per season is also indicated for reference.

## Slide 4
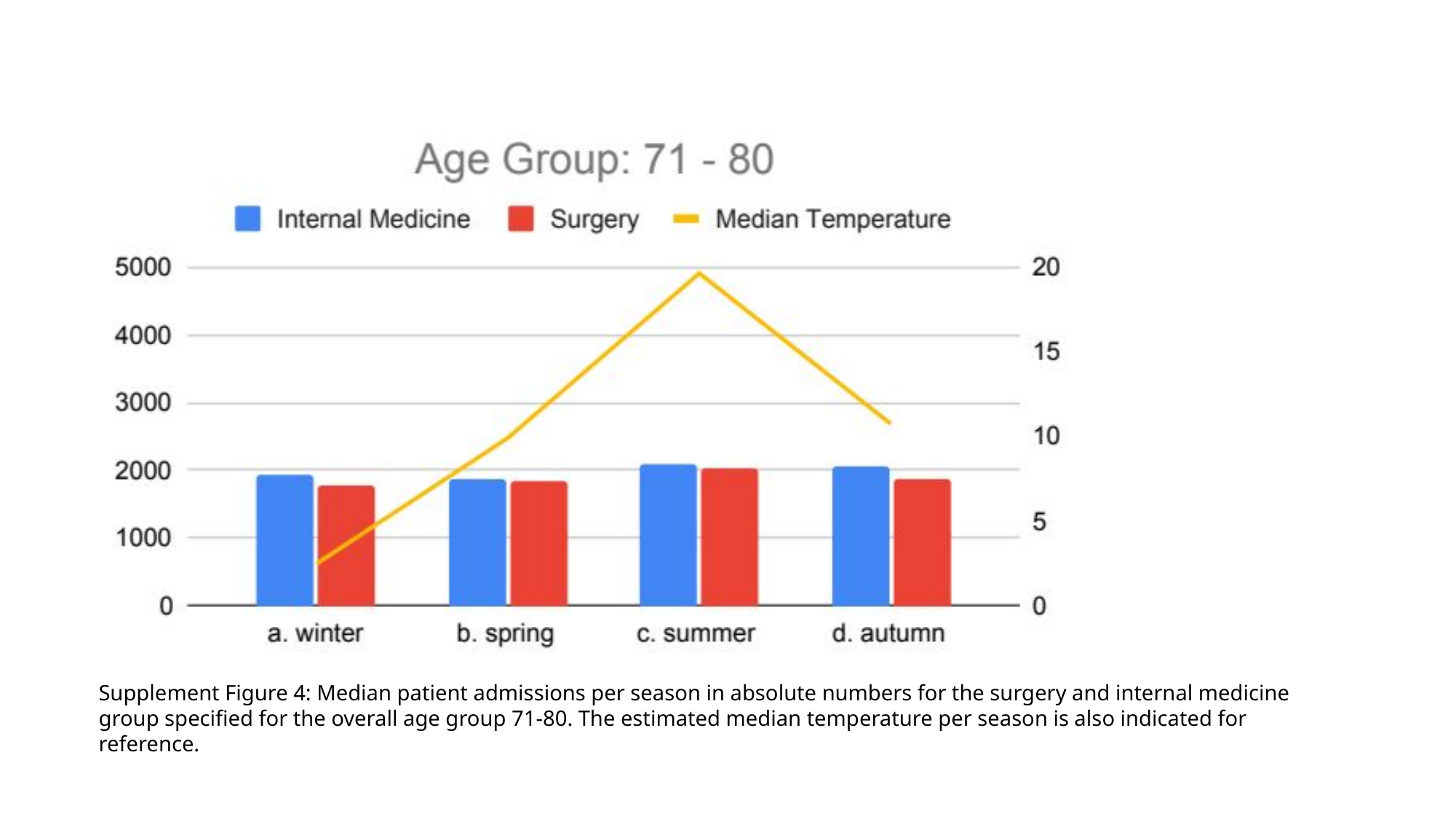

Supplement Figure 4: Median patient admissions per season in absolute numbers for the surgery and internal medicine group specified for the overall age group 71-80. The estimated median temperature per season is also indicated for reference.

## Slide 5
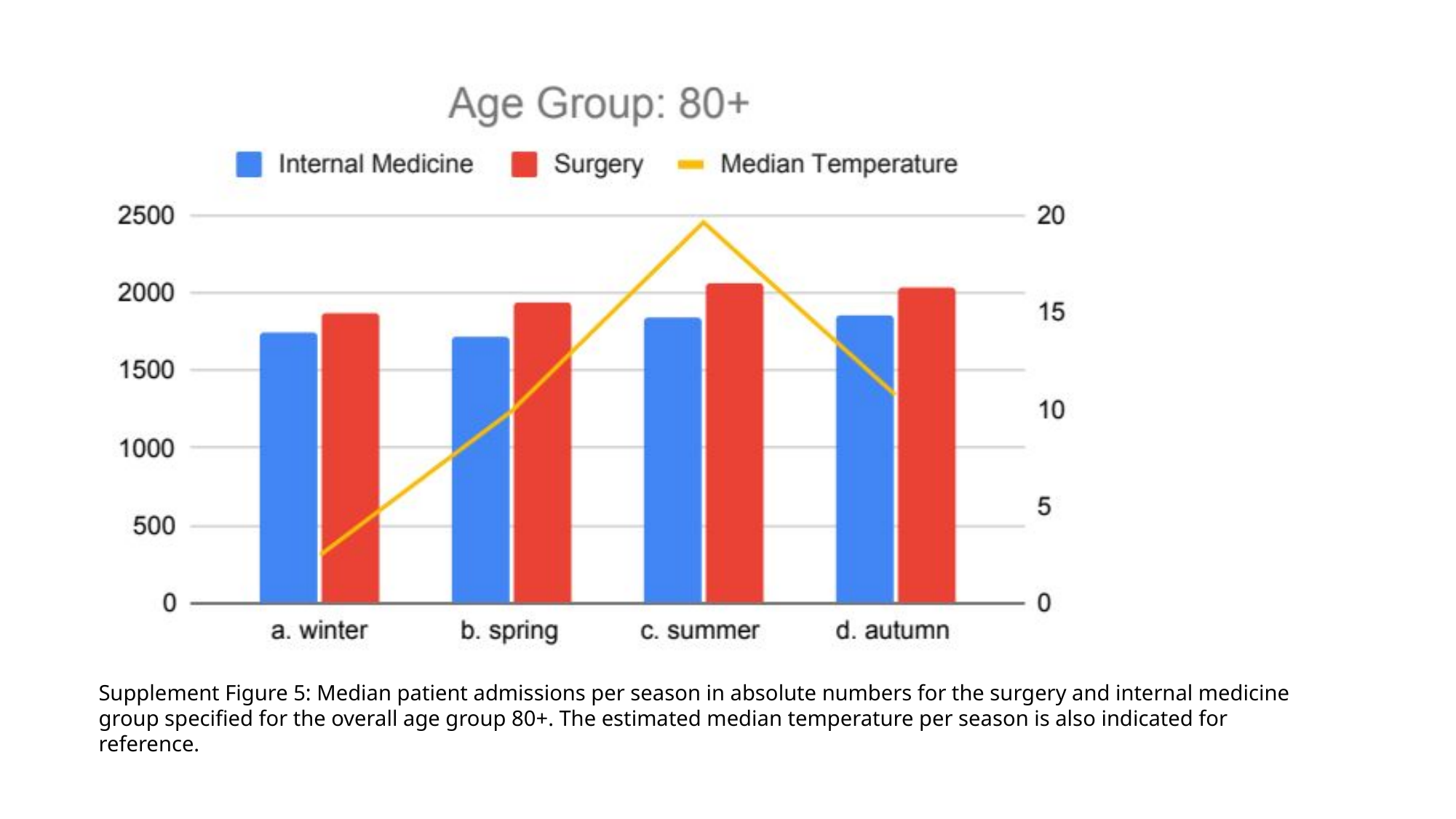

Supplement Figure 5: Median patient admissions per season in absolute numbers for the surgery and internal medicine group specified for the overall age group 80+. The estimated median temperature per season is also indicated for reference.

## Slide 6
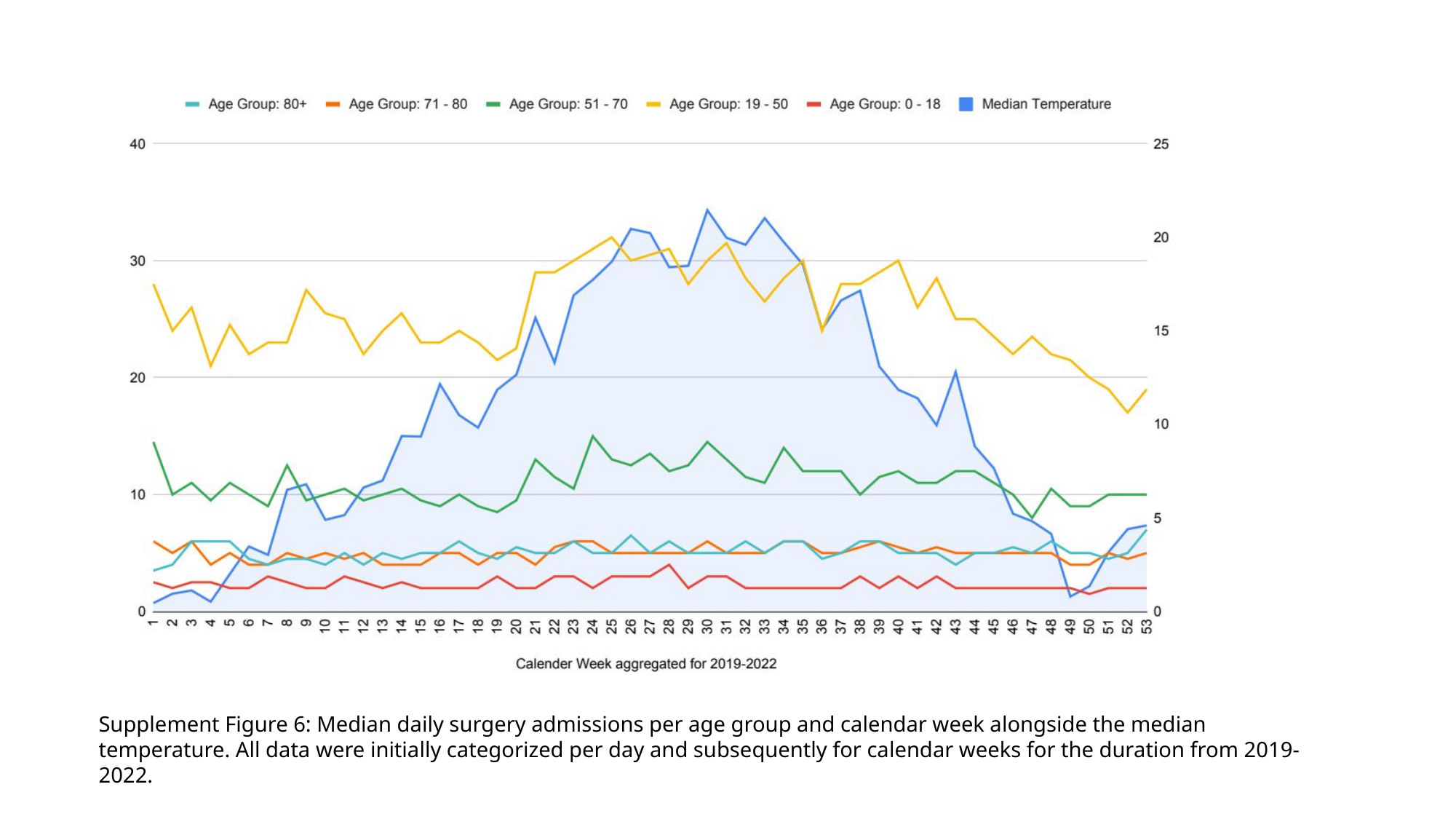

Supplement Figure 6: Median daily surgery admissions per age group and calendar week alongside the median temperature. All data were initially categorized per day and subsequently for calendar weeks for the duration from 2019-2022.

## Slide 7
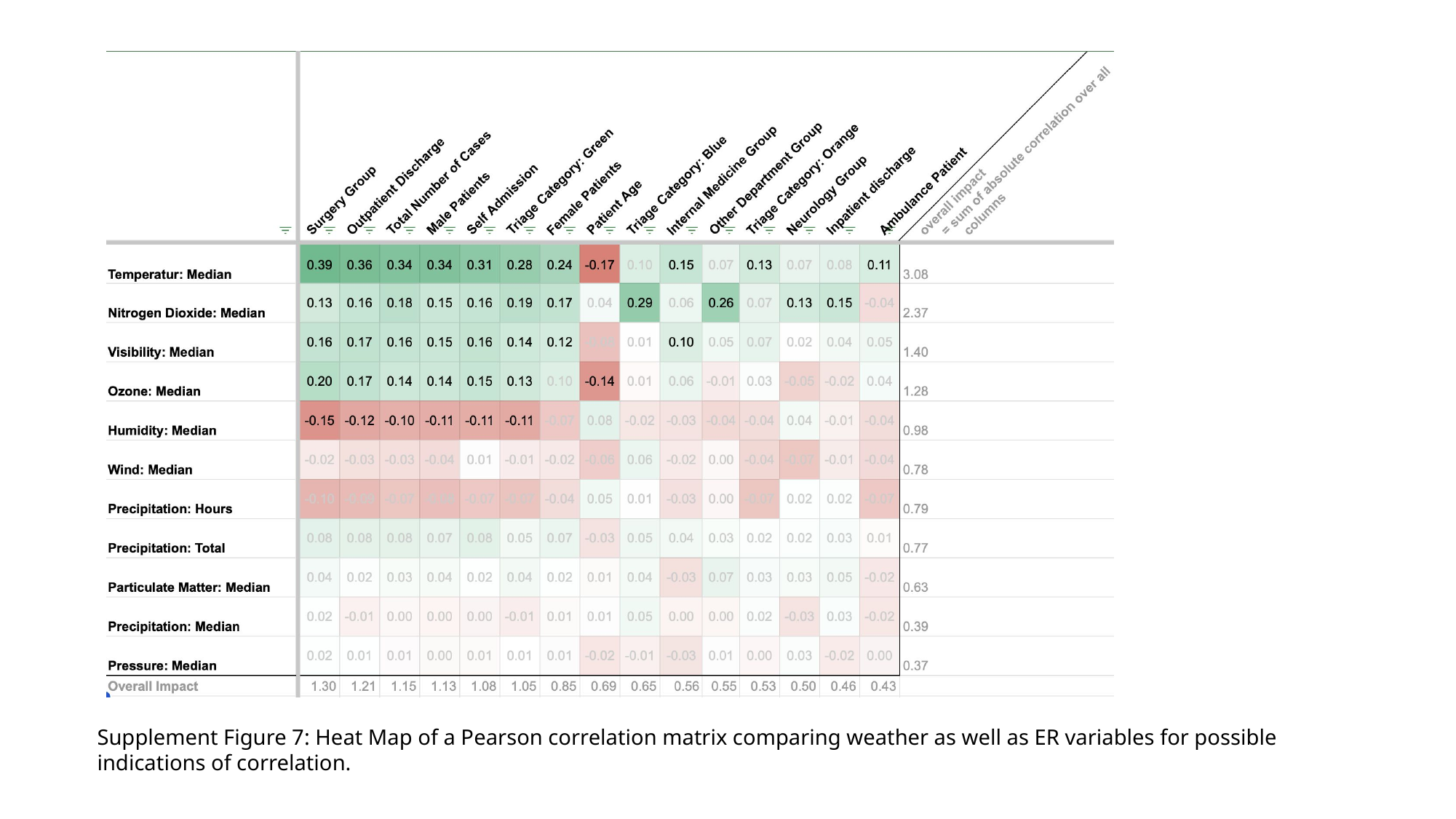

Supplement Figure 7: Heat Map of a Pearson correlation matrix comparing weather as well as ER variables for possible indications of correlation.
